# Autograft dilation after Ross procedure in children and young adults is mitigated by autograft reinforcement: A retrospective MRI study

**DOI:** 10.1038/s41598-025-86953-5

**Published:** 2025-01-22

**Authors:** Raphael Seiler, Robin Stenzel, Viktoria Weixler, Milena Muiznieks, Marina Gürtner, Felix Berger, Titus Kühne, Mi-Young Cho, Joachim Photiadis, Marcus Kelm, Peter Murin

**Affiliations:** 1 Deutsches Herzzentrum der Charité, Department of Congenital Heart Disease – Pediatric Cardiology, Augustenburger Platz 1, 13353 Berlin, Germany; 2 Deutsches Herzzentrum der Charité, Department of Congenital and Pediatric Heart Surgery, Augustenburger Platz 1, 13353 Berlin, Germany; 3 Charité - Universitätsmedizin Berlin, corporate member of Freie Universität Berlin and Humboldt-Universität zu Berlin, Charitéplatz 1, 10117 Berlin, Germany; 4 Deutsches Herzzentrum der Charité, Institute of Computer-assisted Cardiovascular Medicine, Augustenburger Platz 1, 13353 Berlin, Germany; 5 DZHK (German Centre for Cardiovascular Research), partner site Berlin, Potsdamer Str. 58, 10785 Berlin, Germany

**Keywords:** Cardiac MRI, Ross procedure, Congenital heart disease, Aortic disease, Autograft dilation, Autograft reinforcement, Cardiology, Interventional cardiology, Paediatric research, Medical research, Outcomes research

## Abstract

Limited magnetic resonance imaging (MRI) data on autograft dilatation following the Ross procedure in congenital cohorts presents challenges in understanding its evolution and impact on clinical outcomes. This study, spanning from February 2003 to December 2022, included patients under 40 years at the time of the Ross procedure, with MRI follow-ups assessing dimensions at key aortic sites. Among 307 patients, 132 MRIs were analyzed from 76 individuals, revealing that autograft z-scores increase primarily with time post-procedure (Coef. 0.13; 95% CI:0.051–0.216; P = 0.002). Additionally, older patients at the time of surgery showed larger ascending aortic dimensions (Coef. 0.13; 95% CI:0.099–0.165; P = 0.001). Notably, autograft dilation at the sinus of Valsalva significantly predicted higher reintervention risks (HR 1.57; 95% CI:1.21–2.04; P = 0.001). Surgical reinforcement techniques of the autograft, via subcoronary implantation or external support, prevented such dilation (P < 0.001) and mitigated aortic regurgitation. In conclusion, our model predicted autograft dilation over time in patients after Ross procedure, aiding clinicians in making data-driven decisions regarding the optimal timing of the procedure and the selection of the most effective surgical strategy.

## Introduction

The Ross procedure, first described by Donald Ross in 1967^1^, offers a promising alternative to prosthetic valve replacement for infants, children and young adults with congenital aortic valve disease. This procedure offers several advantages, including favorable hemodynamics, obviation of the need for anticoagulation, and lower risks of endocarditis and thromboembolism. Additionally, it allows for the autograft diameter to increase with somatic growth, particularly in young children^2^. Studies have shown excellent survival rates and quality of life following the Ross procedure^3^. However, the placement of the pulmonary autograft under systemic pressure is a known risk factor for the remodeling and dilation of the autograft and the ascending aorta^2,4,5^leading to autograft-related reinterventions, typically in the second decade post-surgery^6^.

Systematic follow-up using multimodal imaging is crucial for the early detection of pathological changes after Ross procedure, facilitating data-driven decision-making. Magnetic resonance imaging (MRI) is increasingly applied in cardiac diseases, especially in valvular heart disease. MRI enables precise quantification of valvular stenosis and regurgitation severity^7^. It also accurately assesses ventricular function and volumes, and provides a detailed evaluation of the thoracic aorta, including geometry, dimensions, distensibility, shear stress, and flow dynamics^8^. These capabilities of MRI are increasingly utilized in the surgical planning of tailored approaches and in the follow-up of adult patients with aortic valve disease^9,10^.

To date, MRI data regarding long-term changes of autograft dimensions within a congenital cohort are limited. Particularly, there is a lack of long-term follow-up MRI data post-Ross procedure that could assist clinicians in optimizing the timing for reinterventions. We aimed to investigate long-term MRI data on both autograft and aortic dimensions, the surgical technique, and the likelihood of potential reinterventions, to develop an age-dependent predictive model for autograft dimensions following the Ross procedure.

## Patients and methods

### Ethical statement

Ethics Committee approval (EA2/080/20) was granted on 27 August 2020 from Charité Ethics Committee, Campus Charité Mitte, Charitéplatz 1, 10,117 Berlin. Informed consent was obtained from all participants and/or their legal guardians. The authors hereby confirm that all research was performed in accordance with relevant guidelines, regulations and the Declaration of Helsinki.

### Study population and design

A single-center, retrospective cohort study was conducted. It included all patients under the age of 40 who underwent the Ross procedure between February 2003 and December 2022 and had at least one postoperative MRI examination. To incorporate MRI datasets from individuals with multiple follow-up visits at varying intervals, each MRI follow-up dataset was treated as a distinct case, characterized by its unique baseline and outcome features.

### Clinical follow-up

Medical records including preoperative data, surgical notes and outpatient reports, were reviewed. Postoperative clinical follow-up data were available for all included cases. Follow up includes mainly patients with a clinical indication for MRI.

### Operative techniques

In our cohort both, the free-root and subcoronary techniques were employed for autograft implantation. From 2003 to 2012, free-root implantation without additional wrapping of the autograft was performed in all cases by a single main surgeon, only aortic annulus was wrapped with a pericardial stripe. Starting in 2013, autograft reinforcement techniques including the modified subcoronary autograft implantation and external wrapping with PTFE membrane or woven polyester prosthesis, were selectively introduced. These methods were intended to either preserve the integrity of the native aortic root or to provide external reinforcement when deemed appropriate. The details of the currently used subcoronary implantation technique were published previously^11^. For right ventricular outflow tract reconstruction, pulmonary homografts were the preferred option when available; in their absence, a range of biological valve substitutes were employed, implanted in a standard manner on a beating heart.

### MRI image acquisition

**Fig. 1:**
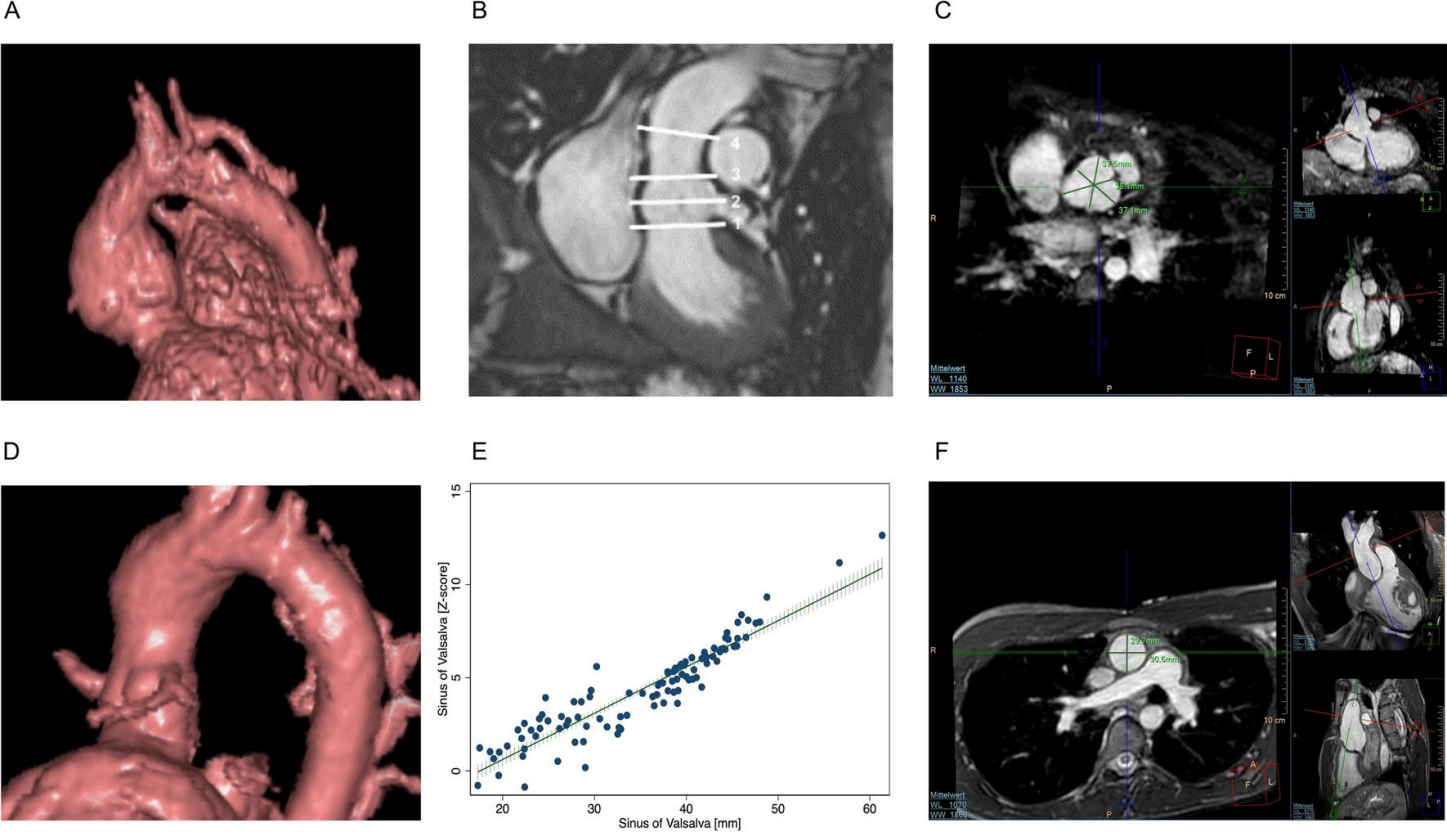
MRI image acquisition *Representative 3D-Reconstruction of autograft after free-root implantation (****A****) and modified subcoronary implantation (****D****) created using ViewForum (R6.3V1L7 SP1; Philips Healthcare). Measured locations across the pulmonary autograft (****B****): 1* = *aortic annulus; 2* = *sinus of Valsalva; 3* = *Sino-tublar junction; 4* = *ascending aorta. Representative 3D whole heart sequence images of sinus of Valsalva (****C****) and ascending aorta (****F****). Sinus of Valsalva diameter in relation to sinus of Valsalva diameter normalized to BSA (z-score) (****E****). MRI* = *Magnetic resonance imaging.*

All MRI examinations were performed using a whole-body 1.5 T CMR system (Achieva R 3.2.2.0, Philips Healthcare, Best, The Netherlands). A standard cardiac MRI protocol was applied (scan duration in total was 9–14 min; Fig. 1):

Standard balanced fast field echo cine imaging. Typical imaging parameters were voxel size 1.80 × 1.70 × 6 mm, reconstructed voxel size 1 × 1 × 6 mm, echo time = 1.2 ms, repetition time = 2.5 ms, flip angle 60°, retrospective cardiac gating, 40 automatically reconstructed cardiac phases.

3D anatomical imaging, acquired at end-diastole. The sequence parameters were adapted to patient’s age and size: acquired voxels by default were 0.66 × 0.66 × 3.2 mm, reconstructed voxels 0.66 × 0.66 × 1.6 mm, repetition time 4.0 ms, echo time 2.0 ms, flip angle 9, number of signal averages 3, navigator gating.

Phase contrast flow measurement: Steady-state free-precession MRI acquisitions were used for anatomical planning. Flow measurements were done perpendicular to each targeted vessel and with a free- breathing phase contrast cine MRI technique.

### MRI image postprocessing

Analysis was performed using View Forum (R6.3V1L7 SP1; Philips Healthcare).

Autograft dimensions were assessed at: Aortic annulus (AA), sinus of Valsalva (SV), sino-tubular junction (STJ) and ascending aorta at level of the pulmonary artery bifurcation (AscA) (Fig. 1B, C, F). These segments were manually selected at end-systole (AA) and end-diastole (SV, STJ and AscA). The imaging planes were positioned perpendicular to the vessel and cross-sectional diameter of AA, STJ as well as AscA were chosen at the shortest distance to minimize minor angulation errors. When we encountered difficulties in delineating the STJ and SV (approx. in 15% of cases with severe dilation) we involved multiple observers to reach a consensus. In cases where delineation was particularly challenging, measurements were made using the best approximation of the anatomical landmarks based on adjacent structures. To assess SV diameter the distances from cusp to commissure from inner edge to inner edge in the annular plane for each cusp were measured, and the three values were averaged^12^ (Fig. 1 C). The 3D-Reconstructions (Fig. 1 A and D) were created using View Forum (R6.3V1L7 SP1; Philips Healthcare), representative cases were selected.

### Z-scores

Raw autograft segment and ascending aorta measurements were normalized to the patient’s body surface area and converted to z-scores, utilizing MRI data from a normal pediatric population for reference^13,14^. The correlation between raw dimensions and z-scores is shown on the example of SV in Fig. 1 E.

### Statistical analysis

The available MRI data in conjunction with demographic, anthropometric and clinical data were used for further statistical analysis. A linear regression model with robust standard errors was fitted, incorporating variables such as age at the time of the Ross procedure, time elapsed since the Ross procedure, age at MRI examination, as well as the operative technique (free-root vs. modified subcoronary autograft implantation/external autograft reinforcement). These factors were assumed to be potential risk factors for autograft dilation. Additionally, Cox proportional hazard models were employed to analyze data related to autograft reinterventions. The dimensions of the autograft and ascending aorta, along with the aforementioned covariates, were included in these models.

In our statistical model, ELASTICNET regularization was utilized. This process began with a model incorporating all covariates of interest. The quality of the resulting models, formed by the sequential removal of each covariate, was evaluated using the models’ Lambda and Bayesian Information Criterion (BIC) and the model’s concordance, or C-statistic^15^. This algorithm continued until no further improvement in the BIC was observed upon the removal of an additional variable or until all variables were excluded. Patient data were extracted and managed using the REDCap® database (Version 12, Vanderbilt University, Nashville, Tennessee, USA). Continuous data are reported as median and interquartile range [IQR; Q25-Q75], unless specified otherwise. Categorical data are presented as frequencies and percentages (%). Statistical analyses were conducted using Stata V.18.0, with P-values < 0.05 were indicative of statistical significance.

## Results

**Fig. 2:**
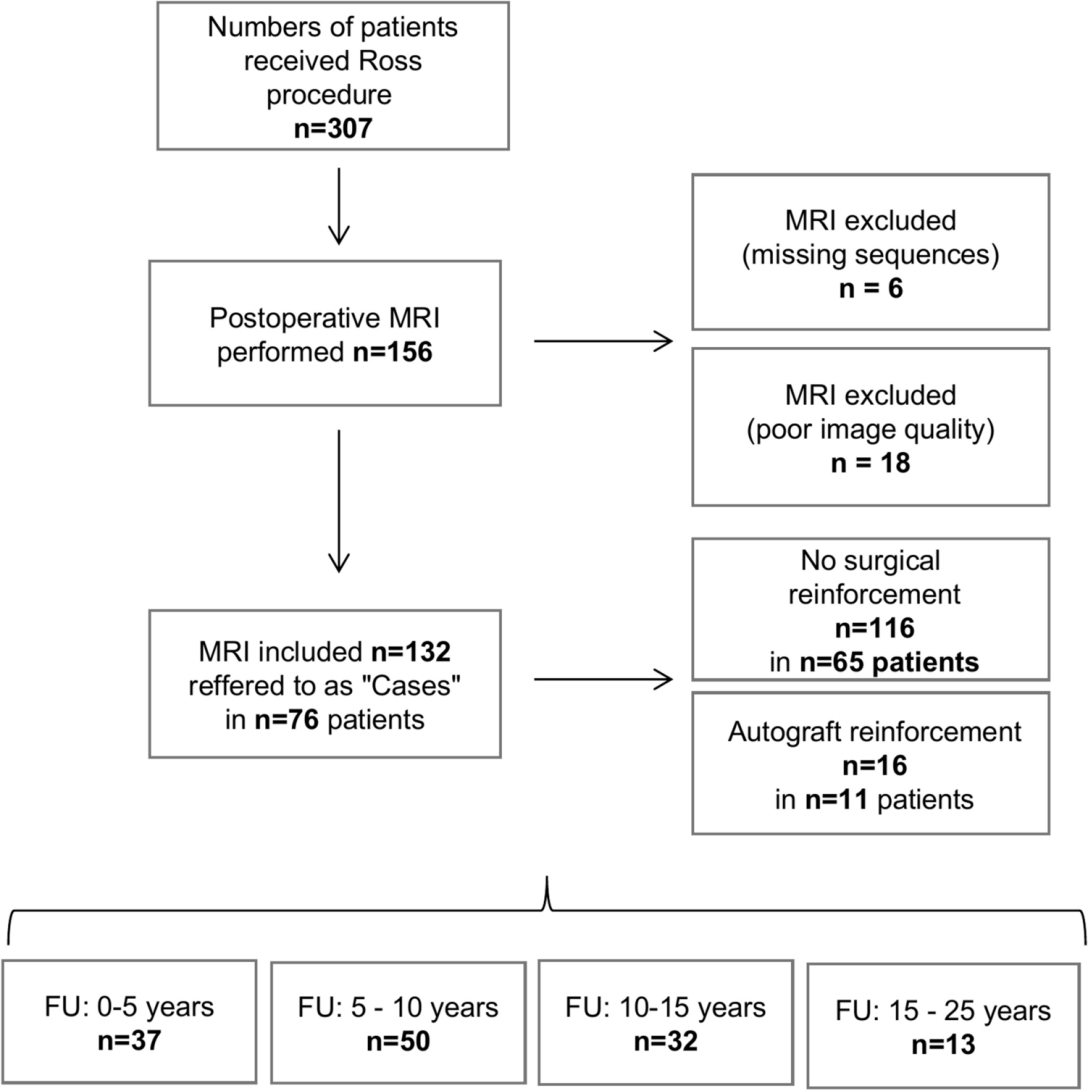
Study flow of participants *FU* = *Follow-up time; MRI* = *Magnetic resonance imaging.*

From February 2003 to December 2022, 307 patients underwent the Ross procedure at our institution, as shown in Fig. 2. The median age at the time of the procedure was 13.6 years [IQR 5.2–30.0]. Among them, 76 patients (27 females; 35.6%, 7 infants; 9.2%) underwent at least one follow-up MRI, totaling 132 follow-up MRI investigations. The median age at the time of MRI was 23 years [IQR 12.8–35.5], with a median interval of 7.9 years [IQR 4.2–12.1] from the Ross procedure to the MRI. Baseline characteristics at the time of MRI examination are detailed in Table 1. Free-root replacement was conducted in 116 cases (87.8%), while autograft reinforcement, either by modified subcoronary implantation (n = 11), PTFE membrane (n = 3), or polyester prosthesis (n = 2), was applied in 16 cases (12.2%). The primary indications for the Ross procedure were combined aortic valve lesion (72.0%), aortic regurgitation (15.2%), and aortic stenosis (12.9%). Prior to the Ross procedure, interventions included aortic valve repair in 47 cases (35.6%), balloon valvuloplasty in 21 (15.9%), a combination of both in 27 (20.5%), while 37 cases (28%) had no previous treatment.

**Table1:** Basic clinical data at MRI examination.

MRI Examinations cases (n)	132
**Patients (n)** Female (n, %) Infants (n, %)	76 *27(35.6)* *7 (9.2)*
**Follow-up interval (years, IQR)**	7.9 [4.2–12.1]
**Age at MRI (years, IQR)**	23 [12.8–35.5]
**Height (cm, IQR)**	170 [145.3–180.0]
**Weight (kg, IQR)**	65.5 [36.3–85.5]
**BSA (m**^**2**^**, IQR)**	1.79 [1.2–2.1]
**BMI (kg/m**^**2**^**, IQR)**	22.6 [17.2–26.1]
**Bicuspid aortic valve (n, %)**	86 (65)
**Age at Ross procedure (years, IQR)**	13.6 [5.2–30.0]
**Initial leading lesion** Aortic stenosis (n, %) Aortic regurgitation (n, %) Combined (n, %)	17 (12.9) 20 (15.2) 95 (72.0)
**Previous treatment** None (n, %) Ballon valvuloplasty (n, %) Aortic valve repair (n, %) Ballon valvuloplasty and aortic valve repair (n, %)	37 (28.0) 21 (15.9) 47 (35.6) 27 (20.5)
**Surgical technique** Free-root (n, %) Autograft reinforcement (n, %) Subcoronary implantation (n, %) PTFE membrane (n, %) Polyester prothesis (n ,%)	116 (87.8) 16 (12.2) *11 (8)* *3 (2)* *2 (2)*
**Autograft reintervention (out of 132 cases)** Due to autograft aneurysm (n, %) Due to aortic regurgitation (n, %) Due to endocarditis (n, %)	16 (12.1) 11 (68.8) 3 (18.8) 2 (12.5)

Continuous variables are given as median with [IQR], categorical variables are given in n (%) IQR = Interquartile range [Q1-Q3], PTFE = Polytetrafluoroethylene).

### Clinical follow-up

Out of the 132 cases studied, 16 (12.1%) required autograft-related reinterventions. The predominant reason for these reinterventions was autograft aneurysm (11 out of 16 cases; 68.8%). AR was the leading indication in three cases (18.8%), and endocarditis in two cases (12.5%). In twelve cases (75%) a mechanical valve replacement was conducted, in three cases (18.8%) a bioprosthetic valve was implanted and in one (6.3%) case the autograft was reconstructed.

During follow-up, two out of 76 patients (2.6%) passed away due to non-cardiac causes.

### Autograft dimensions and aortic regurgitation

**Fig. 3:**
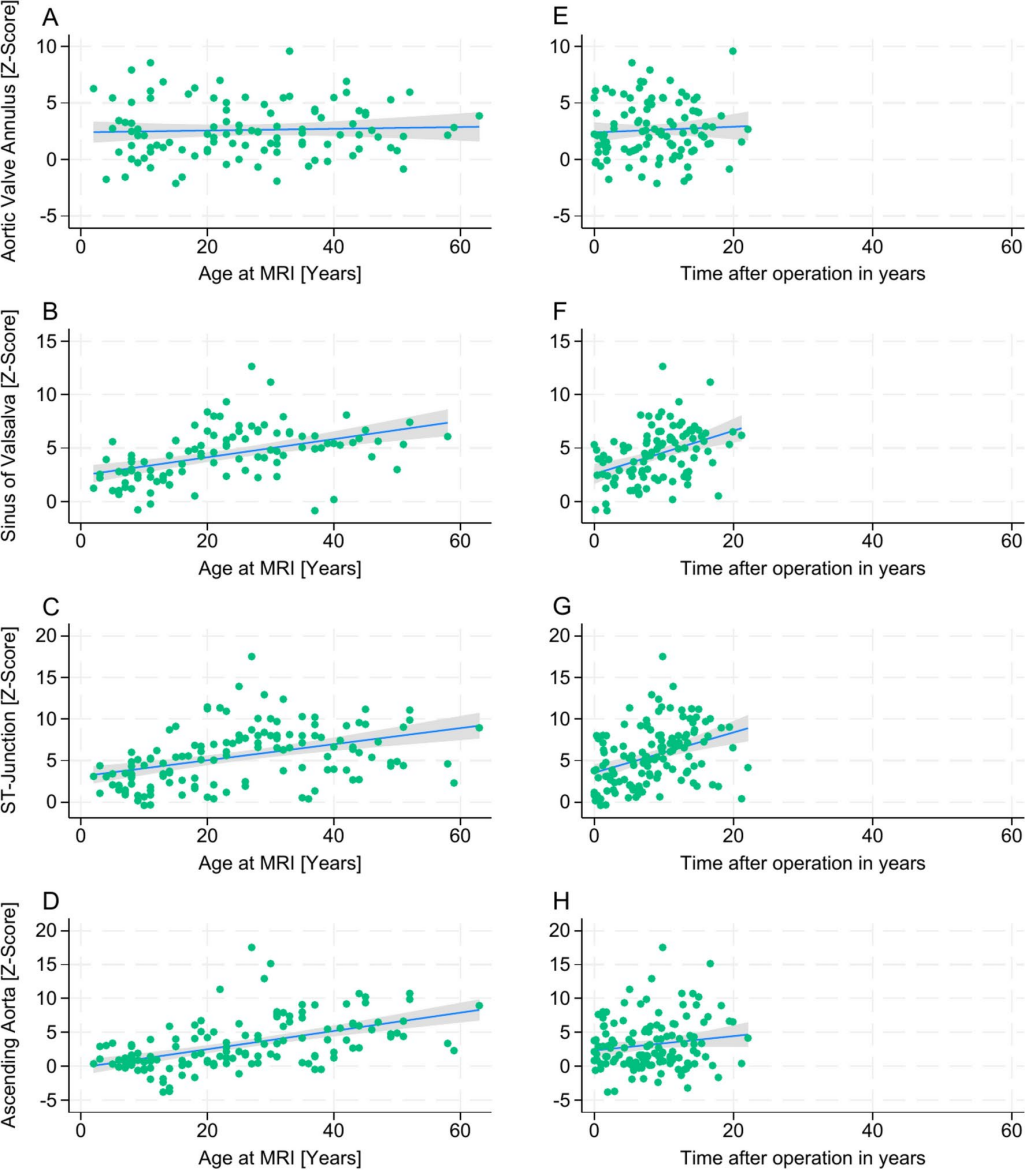
Z-scores of autograft/ aortic dimensions after Ross procedure *Z-scores of aortic segments are plotted against patient age at MRI examination (****A****-****D****) and time after operation in years (****E****–****H****). Aortic valve annulus (****A*** + ***E****); sinus of Valsalva (****B*** + ***F****); ST-junction (****C*** + ***G****); ascending aorta (****D*** + ***H****). MRI* = *Magnetic resonance imaging.*

**Fig. 4:**
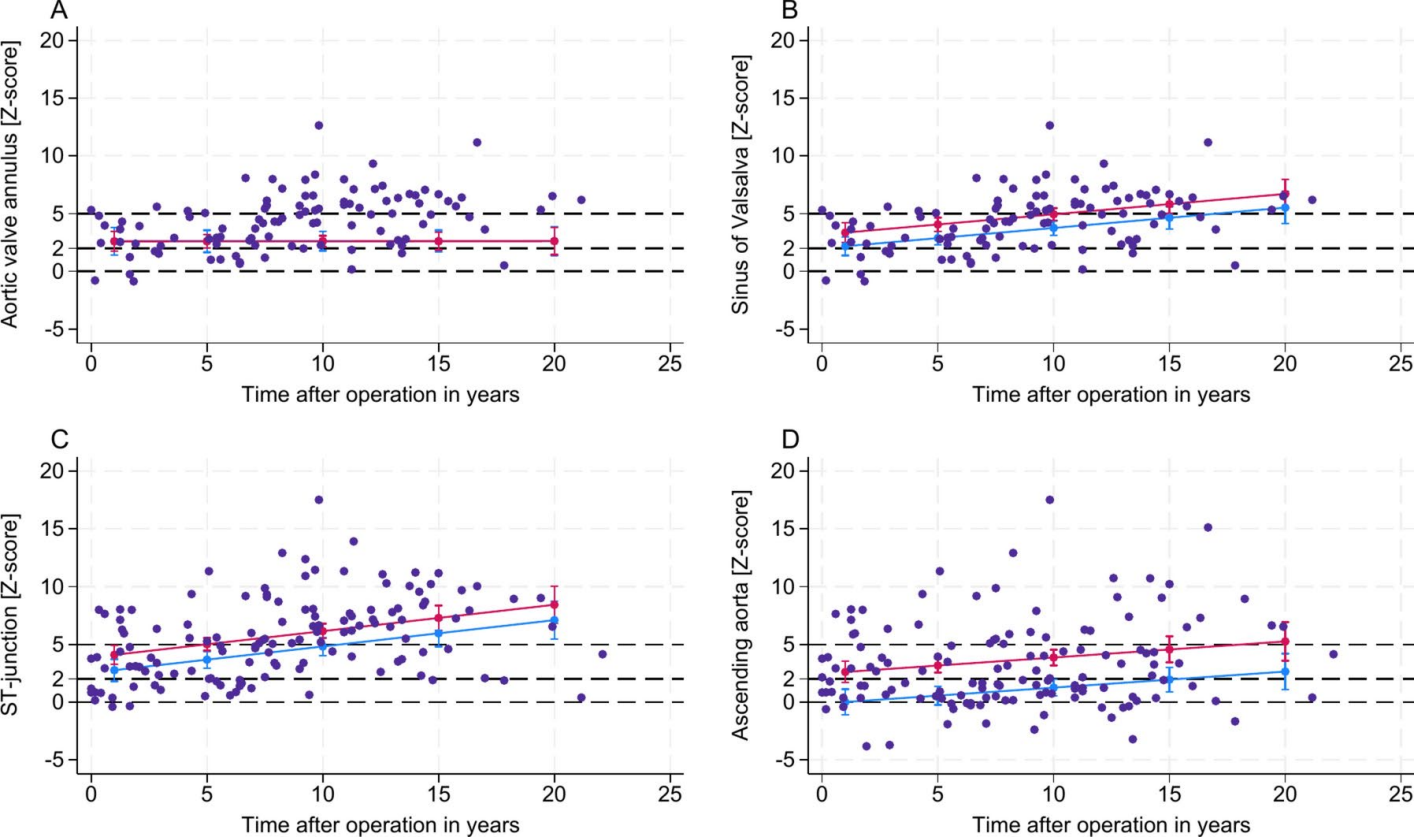
Impact of patients age at time of the Ross procedure and time after Ross procedure on autograft/ aortic dimensions *Z-scores are plotted against elapsed time after operation in years. Predictive margins with 95% CI for z-scores are shown for patient one year of age at time of the Ross procedure in blue and 20 years of age at time of the Ross procedure in red. The cohort data is shown as a scatter plot. Aortic valve annulus (****A****); sinus of Valsalva (****B****); ST-junction (****C****); ascending aorta (****D****).*

Observed autograft dimensions revealed out-of-proportional growth with a great variability across different age groups. Our model revealed that increased z-scores of the SV and the STJ were primarily predicted by the elapsed time post-Ross procedure. The age at the time of the Ross procedure had a minimal impact on the z-scores of SV and STJ over time, as depicted in Figs. 3 and 4. Specifically, each year after the Ross procedure corresponded to an estimated increase in the z-score of + 0.13 per year at SV (95% CI: 0.051–0.216, P = 0.002) and + 0.19 per year at STJ (95% CI: 0.085–0.299, P = 0.001).

In contrast to SV and STJ, for the AscA, the age at the time of the Ross procedure was a significant predictor of increases in z-scores, while the elapsed time post-procedure did not significantly affect AscA dilation, as shown in Figs. 3 and 4. Specifically, an older age at the time of the Ross procedure correlated with higher AscA z-scores, with each additional year of age contributing to an estimated increase of + 0.13 in the z-score (95% CI: 0.099–0.165, P = 0.001). Additionally, dilation of SV, STJ, and AscA was linked to a higher grade of AR (P < 0.001 for SV and AscA; P = 0.002 for STJ), whereas the dimensions of the AA showed no significant impact on the grade of AR.

**Fig. 5:**
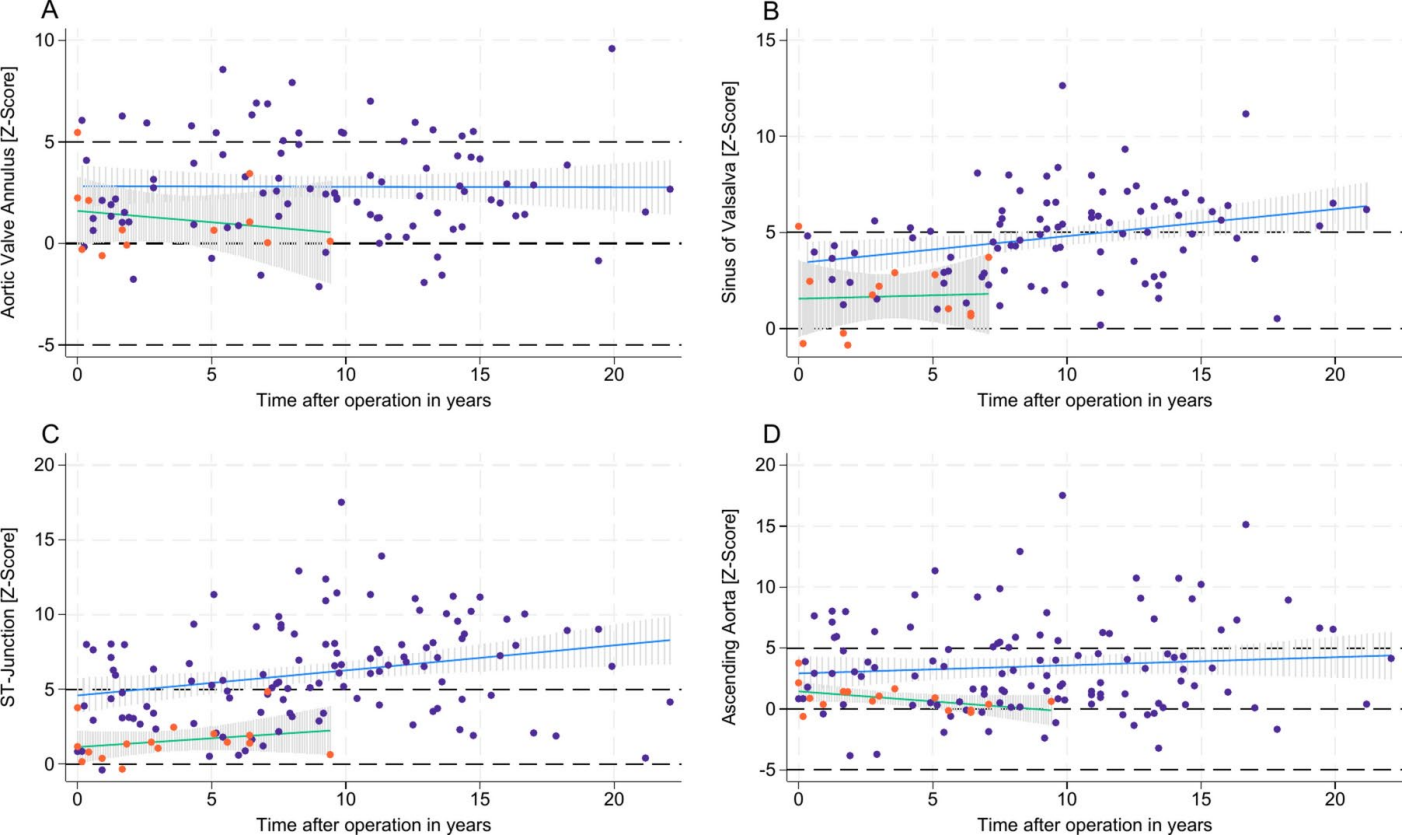
The effect of autograft reinforcement on autograft/ aortic dimensions after Ross procedure over time *Z-scores are plotted against elapsed time after operation in years. Blue dots and blue line representing cases without autograft reinforcement; Orange dots and green line representing cases with autograft reinforcement. Aortic valve annulus (****A****); sinus of Valsalva (****B****); ST-junction (****C****); ascending aorta (****D****). MRI* = *Magnetic resonance imaging.*

**Fig. 6:**
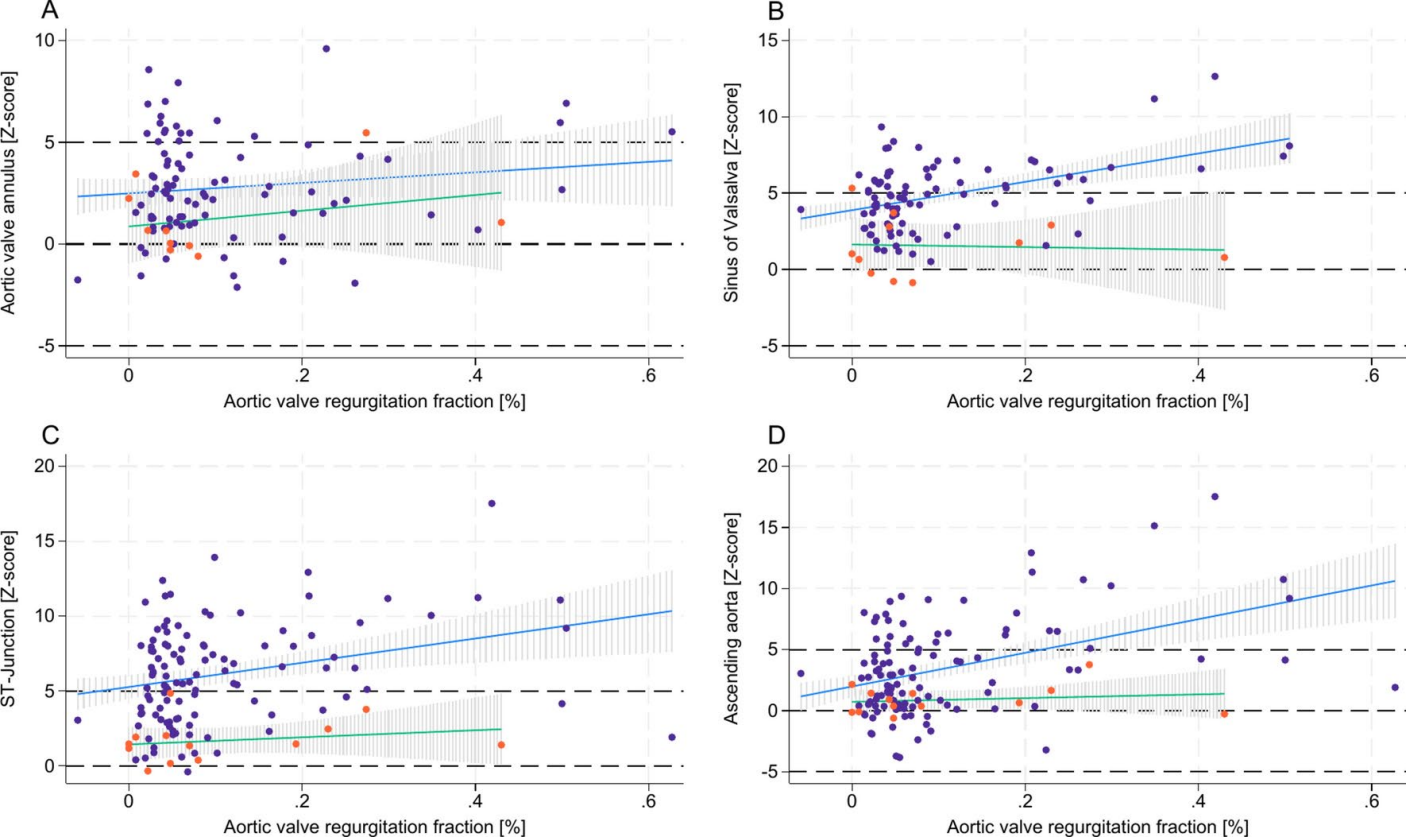
The effect of autograft reinforcement on autograft/ aortic dimensions and its relation to aortic regurgitation *Z-scores are plotted against aortic valve regurgitation fraction (%) for aortic valve annulus (****A****), sinus of Valsalva (****B****), ST-junction (****C****) and ascending aorta (****D****). Blue dots and blue line representing cases without autograft reinforcement; Orange dots and green line representing cases with autograft reinforcement.*

Our model indicated that autograft reinforcement, either through subcoronary implantation or external autograft reinforcement, was associated with reduced z-scores across the SV, STJ, and AscA. The reductions were statistically significant, with respective RMSE (R^2^) values of 2.07 (0.31), 2.95 (0.30), and 3.21 (0.28), and an overall model’s P-value of < 0.001 (Fig. 5). Furthermore, patients who received autograft reinforcement exhibited a markedly lower likelihood of developing a higher grade of AR, with no progression over time, as illustrated in Fig. 6. Taken together, in cases with surgical autograft reinforcement, z-scores for SV, STJ, and AscA remained stable over time. This stability was associated with the absence of AR progression (Fig. 5 and 6).

The AA dimensions were unaffected by the time elapsed post-Ross procedure, the age of patients at the time of the procedure, or the surgical reinforcement of the autograft.

Supplemental Tables 1 and 2 provide detailed scenarios analysing the combined effects of age at the time of the Ross procedure, time elapsed post-procedure, and surgical autograft reinforcement on autograft dimensions.

### Autograft-related reinterventions

**Fig. 7:**
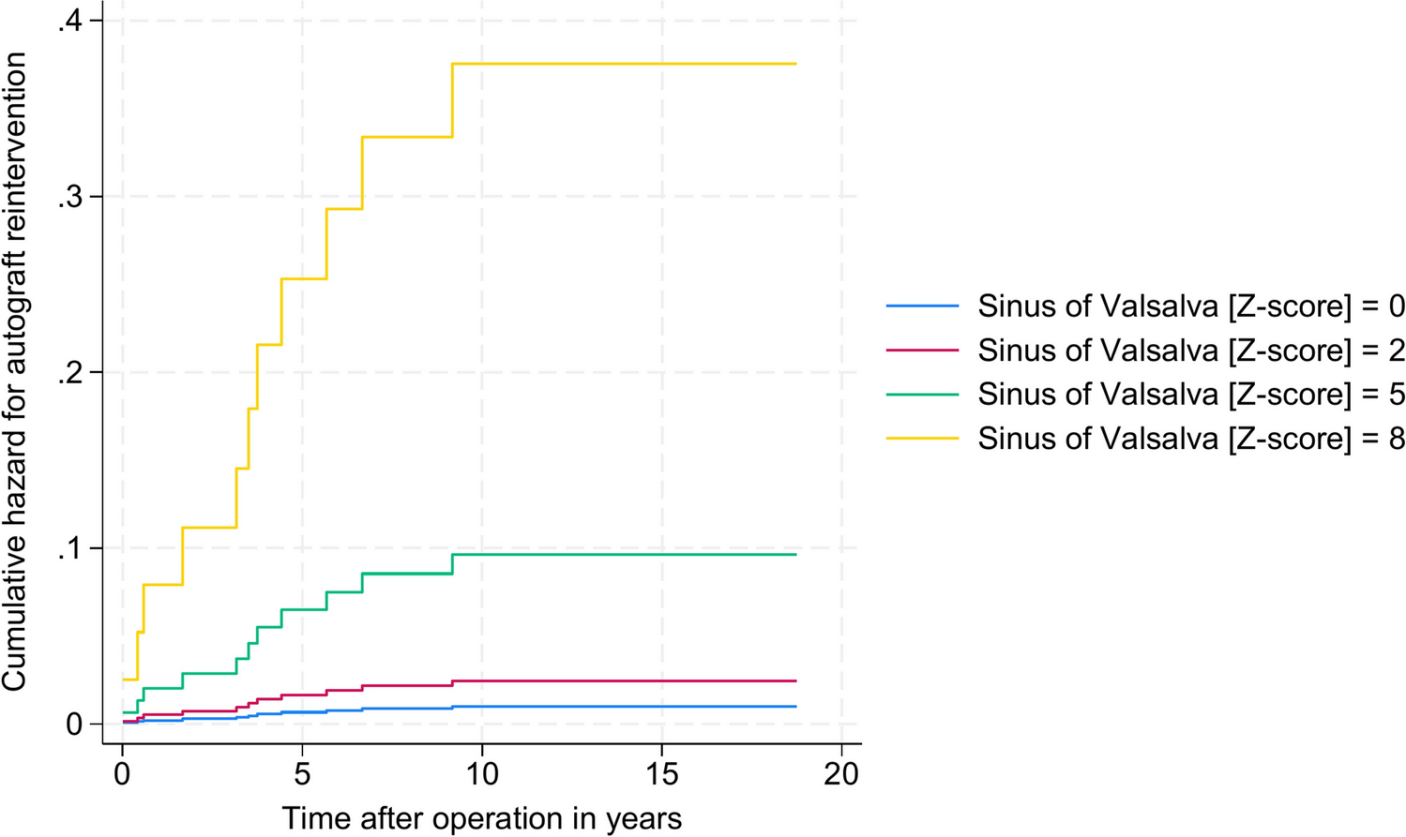
Risk for autograft reintervention *Cumulative hazard for autograft reinterventions depended on sinus of Valsalva z-score and time after operation in years. Z-score of 0, 2, 5, and 8 is represented by different color (blue, red, green, yellow).*

To understand the implications of autograft dilation on reinterventions, a Cox proportional hazard regression model was employed, as previously described. This model revealed an elevated cumulative hazard ratio of 1.57 for autograft reinterventions related to increases in SV z-scores (95% CI: 1.21–2.04, P = 0.001), as shown in Fig. 7. Specifically, a one-unit increase in the SV z-score was associated with a 57% higher risk of autograft reintervention.

## Discussion

The Ross procedure is highly regarded, particularly for children and young adults in need of aortic valve replacement^16^. However, a common late complication is the dilation of the autograft, often associated with progressive aortic regurgitation and subsequent autograft-related reinterventions^3,5,17^. Reports on long-term clinical outcomes are usually limited to transthoracic echocardiographic data regarding autograft dimensions and function. There is a notable gap in the current literature concerning sufficient MRI data for patients post-Ross procedure. This data is crucial for predicting changes in aortic dimensions, assessing autograft function, and evaluating the impact of surgical techniques during long-term follow-up^18^.

In our study, we have employed routine MRI datasets to analyze autograft dimensions normalized by z-scores. This data was then used to create a predictive model to track the progression of autograft dimensions over time and their relationship with different known or suspected risk-factors for autograft dilation. Additionally, we modeled the impact of surgical reinforcement techniques on autograft implantation.

Our analysis of long-term MRI data corroborated the disproportionate progression of autograft dimensions relative to somatic growth. In a robust regression model, we found that the time elapsed post-Ross procedure was the strongest predictor of increased z-scores in the SV and STJ. Furthermore, infants and children who underwent the operation at a younger age exhibited a slightly reduced risk of autograft dilation over time. These findings are in line with previous reported results from pediatric and adult patients after Ross procedure^5,19,20^. This pattern may be primarily attributed to the fact that the pulmonary autograft tends to dilate over time when subjected to systemic pressure, particularly in older children and adults. Systemic circulation pressure is about six times higher than pulmonary circulation pressure immediately after transposition, leading to significantly elevated wall stress in the pulmonary autograft and subsequent dilation^21^. However, infants and younger children’s autograft tissue appears to possess greater adaptability and remodeling capacity to systemic pressure, thereby reducing the extent of autograft dilation^22,23^.

Our study identified patient age at the time of the Ross procedure as a strong predictor for increases in AscA z-scores. Consequently, older patients at the time of surgery showed higher AscA z-scores over time. This trend may be attributed to the prolonged exposure to turbulent flow in the AscA prior to the Ross procedure in older patients, leading to subsequent dilation even after aortic valve replacement. Particularly, patients with an initial bicuspid valve morphology are at an elevated risk for AscA dilation. This increased risk could be due to intrinsic vascular matrix remodeling of the proximal aorta^24^.

As autograft/ AscA dilation is a known risk factor for autograft-related reinterventions, a cox proportional model was fitted to evaluate the risk for autograft-related reinterventions. Our analysis distinctly showed that autograft dilation, particularly at the level of the SV, irrespectively of employed surgical technique, led to an increased risk for reintervention and was associated with a higher grade of AR. Autograft dilation alters the entire valve geometry, impairing the coaptation of valve leaflets, thus leading to the progressive worsening of AR. Additionally, the turbulent flow induced by AR in the aortic segments may contribute significantly to the development of autograft/AscA dilation. Therefore, while our data established a clear association between autograft dilation and AR, it remains inconclusive whether AR is a result of autograft/AscA dilation or vice versa. Comparable to our findings, David et al. reported that autograft dilation was associated with a higher rate of autograft-related reinterventions in an adult population and was linked to aortic regurgitation^17^. Notably, the aortic annulus in the entire cohort demonstrated no dilation over time or association to AR, since AA was already supported before the year 2012 by a pericardial strip, to reinforce the autograft annulus. Further, while out of proportion dilation was prevented, in infants and children all segments increased with somatic growth.

Autograft dilation may represent a modifiable risk factor, and its prevention could enhance long-time outcome after Ross procedure. Techniques like subcoronary autograft implantation or external prosthetic support may preserve autograft integrity, thereby preventing dilation^11,25^.

In our cohort, surgical autograft reinforcement was associated with stable z-scores at SV, STJ, and AscA and with the absence of AR progression, irrespective of the time elapsed after the Ross procedure or the age at surgery. In contrast, AR strongly correlated with autograft dilation in non-reinforced cases, suggesting that preventing autograft dilation via autograft reinforcement reduces the grade of the AR. To account for this, AR was excluded as a direct predictor in our regression models, prioritising the autograft dimension as the primary determinant of the risk for autograft reintervention. These findings underscore the clinical importance of preventing autograft dilation to reduce the risk for autograft reinterventions and limit AR progression.

Nevertheless, in order to ascertain whether the documented protection against autograft dilation through surgical reinforcement reduces the incidence of autograft-related reintervention at a individual patient level, the case-based methodology employed in the current study is deemed unsuitable. The propensity for overinterpretation of individual outcomes within a cohort containing only a small number of patients with surgical reinforcement notably diminishes the generalizability of findings. Hence, additional well-powered studies are warranted to proof that the prevention of autograft dilation following the Ross operation through surgical reinforcement effectively diminishes the necessity for autograft-related reinterventions. Furthermore, incorporating advanced techniques such as 4D flow MRI in future studies could enhance the identification of patients at higher risk for autograft dilation by detecting abnormal flow patterns and providing insights into the mechanisms of autograft remodeling. These techniques may also improve surgical strategies to mitigate adverse hemodynamic effects and the progression of AR.

Based on our data on autograft dilation after the Ross procedure, we recommend performing a baseline MRI within the first year after surgery to establish autograft dimensions and function. In patients without significant autograft dilation or AR, MRI examinations should be repeated every 2–3 years. If autograft dilation or AR progresses, more frequent imaging (e.g., annually) may be warranted. Supplemental Tables 1 and 2 outline various scenarios combining patient age at the time of the Ross procedure and the time elapsed since surgery, both with and without surgical autograft reinforcement. These scenarios may help assess the risk of autograft dilation progression over time based on patient-specific factors, including age, timing of MRI, and the surgical technique initially employed. Consequently, these findings provide a data-driven framework for optimising the frequency of MRI examinations in this patient cohort.

## Limitations

The present study’s single-center nature limits its generalizability to all post-Ross procedure patients. Its retrospective design and the inclusion criterion of at least one MRI examination might have introduced selection bias, as included patients had an indication for MRI. Furthermore, due to the retrospective nature of this study and incomplete data on medication dosages, we did not include medication use at the time of MRI as a variable in our analysis. Future studies should consider the potential effects of medications, such as beta-blockers, on stroke volumes and autograft dilation. In cases with severe dilation, precise delineation of aortic levels—particularly between the SV and STJ—was occasionally more complex, addressed by applying standardised measurement protocols and involving multiple observers to reach consensus where necessary.

To maximize data utilization, we included as many MRI examinations as possible, regardless of the time of assessment. This necessitated a case-based approach for modeling outcomes, potentially leading to overestimations in cases with multiple follow-up MRIs. Additionally, while robust regression modeling offers resilience to outliers, it has limitations, such as the assumption of linearity, the challenge of small sample sizes, and the presence of multiple outliers. Within the context of our cohort’s composition and the variables involved, this modeling approach was deemed appropriate, though limitations remain. Furthermore, the relatively low prevalence of reinterventions and extended periods without autograft-related reinterventions in many patients suggest that additional risk factors might emerge with increased prevalence. Lastly, the small number of patients receiving autograft reinforcement through modified subcoronary implantation or external prosthetic support limits the robustness of these findings, which may evolve as patient numbers increase. Additionally, mean follow up times in patients with autograft reinforcement were lower than in those without autograft reinforcement, however we included both follow up time and autograft reinforcement as covariates within the model.

## Conclusions

Our MRI data-based model successfully predicted autograft dimensions over time, incorporating the initial surgical technique, patient age at the time of the Ross procedure, and time elapsed post-procedure. Autograft dilation significantly increases the risk of reintervention; however, autograft reinforcement techniques appear to prevent dilation across aortic segments, potentially improving long-term outcomes after the Ross procedure. The z-score prediction model we developed can aid clinicians in monitoring autograft dimensions to assess the risk of autograft-related reintervention over time. This supports data-driven decision-making and timely intervention, aiming to preserve the function of the autologous autograft valve in patients with congenital aortic valve diseases. To substantiate these findings, larger multi-institutional studies with structured long-term MRI follow-up are essential.

## Supplementary Information

Supplementary Information.

## Data Availability

The data that support the findings of this study are available on request from the corresponding author, RS. The data are not publicly available since containing information that could compromise the privacy of research participants.

## Abbreviations

AA: Aortic annulus
AscA: Ascending aorta
AR: Aortic regurgitation
CI: Confidence interval
HR: Hazard ratio
IQR: Interquartile range
MRI: Magnetic resonance imaging
RSME: Root-mean-square deviation
STJ: Sino-tubular junction
SV: Sinus of Valsalva

## References

[1] Ross, D. N. Replacement of aortic and mitral valves with a pulmonary autograft. *Lancet* **290**, 956–958 (1967). 10.1016/s0140-6736(67)90794-5 4167516

[2] Etnel, J. R. G. et al. The ross procedure: A systematic review, meta-analysis, and microsimulation. *Circ. Cardiovasc. Qual. Outcomes* **11**, e004748 (2018). 30562065 10.1161/CIRCOUTCOMES.118.004748

[3] Aboud, A. et al. Long-term outcomes of patients undergoing the ross procedure. *J. Am. Coll. Cardiol.* **77**, 1412–1422 (2021). 33736823 10.1016/j.jacc.2021.01.034

[4] Schwartz, M. L., Gauvreau, K., Del Nido, P., Mayer, J. E. & Colan, S. D. Long-term predictors of aortic root dilation and aortic regurgitation after arterial switch operation. *Circulation*10.1161/01.CIR.0000138392.68841.d3 (2004). 15364851 10.1161/01.CIR.0000138392.68841.d3

[5] Pasquali, S. K. et al. The relationship between neo-aortic root dilation, insufficiency, and reintervention following the ross procedure in infants, children, and young adults. *J. Am. Coll. Cardiol.* **49**, 1806–1812 (2007). 17466232 10.1016/j.jacc.2007.01.071

[6] Charitos, E. I. et al. Long-term results of 203 young and middle-aged patients with more than 10 years of follow-up after the original subcoronary ross operation. *Ann. Thorac. Surg.* **93**, 495–502 (2012). 22197618 10.1016/j.athoracsur.2011.10.017

[7] Tzolos, E., Andrews, J. P. & Dweck, M. R. Aortic valve stenosis—multimodality assessment with PET/CT and PET/MRI. *Br. J. Radiol.* **93**, 20190688 (2020). 31647323 10.1259/bjr.20190688 PMC7465843

[8] Ghorbani, N. et al. Impact of valve morphology, hypertension and age on aortic wall properties in patients with coarctation: A two-centre cross-sectional study. *BMJ Open* **10**, e034853 (2020). 32213521 10.1136/bmjopen-2019-034853 PMC7170596

[9] Nordmeyer, S. et al. Flow-sensitive four-dimensional cine magnetic resonance imaging for offline blood flow quantification in multiple vessels: A validation study. *J. Magn. Reson. Imaging* **32**, 677–683 (2010). 20815066 10.1002/jmri.22280

[10] Nordmeyer, S. et al. Circulatory efficiency in patients with severe aortic valve stenosis before and after aortic valve replacement. *J. Cardiovasc. Magn. Reson.* **23**, 15 (2021). 33641670 10.1186/s12968-020-00686-0 PMC7919094

[11] Murin, P. et al. Subcoronary Ross/Ross–Konno operation in children and young adults: Initial single-centre experience. *Eur. J. Cardiothorac. Surg.* **59**, 226–233 (2021). 33141218 10.1093/ejcts/ezaa307

[12] Tomii, D. et al. Sinus of valsalva dimension and clinical outcomes in patients undergoing transcatheter aortic valve implantation. *Am. Heart J.* **244**, 94–106 (2022). 34788603 10.1016/j.ahj.2021.11.004

[13] Kaiser, T., Kellenberger, C. J., Albisetti, M., Bergsträsser, E. & Buechel, E. R. V. Normal values for aortic diameters in children and adolescents – assessment in vivo by contrast-enhanced CMR-angiography. *J. Cardiovasc. Magn. Reson.* **10**, 56 (2008). 19061495 10.1186/1532-429X-10-56 PMC2615773

[14] Kawel-Boehm, N. et al. Reference ranges (“normal values”) for cardiovascular magnetic resonance (CMR) in adults and children: 2020 update. *J. Cardiovasc. Magn. Reson.* **22**, 87 (2020). 33308262 10.1186/s12968-020-00683-3 PMC7734766

[15] Burnham, K. P. & Anderson, D. R. Multimodel inference: Understanding AIC and BIC in model selection. *Sociol. Methods Res.* **33**, 261–304 (2004).

[16] Yokoyama, Y. et al. Ross procedure versus mechanical versus bioprosthetic aortic valve replacement: A network meta-analysis. *J. Am. Heart Assoc.* **12**, e8066 (2023). 36565200 10.1161/JAHA.122.027715 PMC9973571

[17] David, T. E., David, C., Woo, A. & Manlhiot, C. The Ross procedure: Outcomes at 20 years. *J. Thorac. Cardiovasc. Surg.* **147**, 85–93 (2014). 24084276 10.1016/j.jtcvs.2013.08.007

[18] Drullinsky, D. et al. Four-dimensional magnetic resonance after ross procedure for unicuspid aortic valve. *Circ. Cardiovasc. Imaging* **14**, e011500 (2021). 33877873 10.1161/CIRCIMAGING.120.011500

[19] Grotenhuis, H. B. et al. Aortic root dysfunctioning and its effect on left ventricular function in Ross procedure patients assessed with magnetic resonance imaging. *Am. Heart J.* **152**(975), e1-975.e8 (2006). 17070172 10.1016/j.ahj.2006.06.038

[20] Hörer, J. et al. Neoaortic root diameters and aortic regurgitation in children after the ross operation. *Ann. Thorac. Surg.* **88**, 594–600 (2009). 19632419 10.1016/j.athoracsur.2009.04.077

[21] Xuan, Y. et al. Wall stresses of early remodeled pulmonary autografts. *J. Thorac. Cardiovasc. Surg.* **164**, 1728–1738 (2022). 34538420 10.1016/j.jtcvs.2021.08.058 PMC8882694

[22] Ivanov, Y. et al. Strategies to minimise need for prosthetic aortic valve replacement in congenital aortic stenosis—value of the ross procedure. *Semin. Thorac. Cardiovasc. Surg.* **32**, 509–519 (2020). 32061889 10.1053/j.semtcvs.2020.02.015

[23] Nelson, J. S. et al. Long-term survival and reintervention after the ross procedure across the pediatric age spectrum. *Ann. Thorac. Surg.* **99**, 2086–2095 (2015). 25921260 10.1016/j.athoracsur.2015.02.068

[24] Debl, K. et al. Dilatation of the ascending aorta in bicuspid aortic valve disease: a magnetic resonance imaging study. *Clin. Res. Cardiol.* **98**, 114–120 (2009). 19083040 10.1007/s00392-008-0731-0

[25] Charitos, E. I. et al. Autograft reinforcement to preserve autograft function after the ross procedure: a report from the german-dutch ross registry. *Circulation*10.1161/CIRCULATIONAHA.108.843391 (2009). 19752360 10.1161/CIRCULATIONAHA.108.843391

