# Supplementary material for: Autograft dilation after Ross procedure in children and young adults is mitigated by autograft reinforcement: A retrospective MRI study
Source: Sci Rep. 2025 Jan 22;15:2813. doi: 10.1038/s41598-025-86953-5 (PMC11754628; doi:10.1038/s41598-025-86953-5)
Supplement: Supplementary file 1 — Supplementary Information. [file 41598_2025_86953_MOESM1_ESM.docx]

Supplementary material

|  | **Supplemental table 1**. Predicted z-scores dependent on age at Ross procedure and time after operation without surgical reinforcement. Median z-scores; (95% confidence interval). z-score < 4: green boxes; z-score > 4 and < 6: yellow boxes; z-score > 6: red boxes. | | | | | |
| --- | --- | --- | --- | --- | --- | --- |
|  |  | **Time after Ross procedure** | | | | |
|  | **Age at Ross procedure** | **1 year** | **5 years** | **10 years** | **15 years** | **20 years** |
| **Sinus of Valsalva** | **1 year** | 2.79 (2.04-3.54) | 3.32 (2.72-3.93) | 3.99 (3.34-4.65) | 4.66 (3.75-5.57) | 5.33 (4.07-6.58) |
|  | **5 years** | 3.03 (2.32-3.74) | 3.56 (3.03-4.1) | 4.23 (3.67-4.79) | 4.9 (4.07-5.73) | 5.56 (4.38-6.75) |
|  | **10 years** | 3.32 (2.62-4.02) | 3.86 (3.37-4.34) | 4.52 (4.05-5.0) | 5.19 (4.44-5.94) | 5.86 (4.75-6.97) |
|  | **15 years** | 3.62 (2.89-4.34) | 4.15 (3.66-4.65) | 4.82 (4.38-5.26) | 5.49 (4.79-6.18) | 6.15 (5.1-7.21) |
|  | **20 years** | 3.91 (3.12-4.71) | 4.45 (3.89-5.01) | 5.11 (4.64-5.59) | 5.78 (5.09-6.47) | 6.45 (5.42-7.48) |
|  | **25 years** | 4.21 (3.32-5.1) | 4.74 (4.08-5.41) | 5.41 (4.85-5.97) | 6.08 (5.35-6.8) | 6.74 (5.71-7.78) |
|  | **30 years** | 4.5 (3.5-5.51) | 5.04 (4.24-5.83) | 5.7 (5.03-6.38) | 6.37 (5.58-7.17) | 7.04 (5.97-8.11) |
|  | **35 years** | 4.8 (3.66-5.93) | 5.33 (4.4-6.27) | 6.0 (5.18-6.82) | 6.67 (5.77-7.56) | 7.33 (6.21-8.46) |
|  | **40 years** | 5.09 (3.82-6.37) | 5.63 (4.54-6.71) | 6.29 (5.33-7.26) | 6.96 (5.95-7.97) | 7.63 (6.42-8.84) |
| **ST-junction** | **1 year** | 3.38 (2.34-4.42) | 4.15 (3.33-4.96) | 5.11 (4.3-5.91) | 6.07 (4.97-7.16) | 7.03 (5.5-8.55) |
|  | **5 years** | 3.65 (2.68-4.63) | 4.42 (3.69-5.15) | 5.38 (4.66-6.1) | 6.34 (5.31-7.38) | 7.3 (5.82-8.78) |
|  | **10 years** | 4.0 (3.08-4.92) | 4.77 (4.11-5.42) | 5.73 (5.08-6.37) | 6.68 (5.7-7.67) | 7.64 (6.2-9.09) |
|  | **15 years** | 4.34 (3.45-5.23) | 5.11 (4.5-5.72) | 6.07 (5.46-6.67) | 7.03 (6.07-7.99) | 7.99 (6.56-9.42) |
|  | **20 years** | 4.69 (3.79-5.58) | 5.45 (4.83-6.08) | 6.41 (5.8-7.03) | 7.37 (6.4-8.34) | 8.33 (6.89-9.77) |
|  | **25 years** | 5.03 (4.09-5.97) | 5.8 (5.12-6.48) | 6.76 (6.08-7.43) | 7.72 (6.71-8.73) | 8.68 (7.21-10.14) |
|  | **30 years** | 5.37 (4.37-6.38) | 6.14 (5.37-6.91) | 7.1 (6.33-7.87) | 8.06 (6.99-9.13) | 9.02 (7.51-10.53) |
|  | **35 years** | 5.72 (4.62-6.81) | 6.49 (5.6-7.37) | 7.45 (6.56-8.33) | 8.41 (7.24-9.57) | 9.37 (7.79-10.94) |
|  | **40 years** | 6.06 (4.86-7.26) | 6.83 (5.81-7.85) | 7.79 (6.77-8.81) | 8.75 (7.48-10.02) | 9.71 (8.05-11.36) |
| **Asceding aorta** | **1 year** | 0.51 (-0.61-1.63) | 0.92 (0.08-1.76) | 1.43 (0.67-2.19) | 1.94 (0.92-2.97) | 2.45 (0.99-3.91) |
|  | **5 years** | 1.04 (-0.15-2.09) | 1.45 (0.68-2.22) | 1.96 (1.26-2.66) | 2.47 (1.48-3.46) | 2.98 (1.54-4.43) |
|  | **10 years** | 1.7 (0.7-2.69) | 2.11 (1.4-2.81) | 2.62 (1.97-3.27) | 3.13 (2.15-4.1) | 3.64 (2.19-5.08) |
|  | **15 years** | 2.35 (1.4-3.31) | 2.76 (2.09-3.43) | 3.27 (2.63-3.91) | 3.79 (2.8-4.77) | 4.3 (2.84-5.76) |
|  | **20 years** | 3.01 (2.07-3.96) | 3.42 (2.74-4.1) | 3.93 (3.26-4.6) | 4.44 (3.42-5.47) | 4.96 (3.46-6.45) |
|  | **25 years** | 3.67 (2.7-4.64) | 4.08 (3.36-4.8) | 4.59 (3.85-5.33) | 5.1 (4.02-6.19) | 5.61 (4.06-7.16) |
|  | **30 years** | 4.33 (3.31-5.34) | 4.74 (3.94-5.54) | 5.25 (4.41-6.09) | 5.76 (4.59-6.93) | 6.27 (4.65-7.89) |
|  | **35 years** | 4.99 (3.9-6.07) | 5.4 (4.49-6.3) | 5.91 (4.96-6.86) | 6.42 (5.16-7.68) | 6.93 (5.23-8.63) |
|  | **40 years** | 5.65 (4.47-6.82) | 6.05 (5.03-7.08) | 6.57 (5.49-7.65) | 7.08 (5.7-8.45) | 7.59 (5.8-9.38) |

|  | **Supplemental table 2**. Predicted z-scores dependent on age at Ross procedure and time after operation with surgical reinforcement. Median z-scores; (95% confidence interval). z-score < 4: green boxes; z-score > 4 and < 6: yellow boxes; z-score > 6: red boxes. | | | | | |
| --- | --- | --- | --- | --- | --- | --- |
|  |  | **Time after Ross procedure** | | | | |
|  | **Age at Ross procedure** | **1 year** | **5 years** | **10 years** | **15 years** | **20 years** |
| **Sinus of Valsalva** | **1 year** | 0.81 (-0.29-1.91) | 1.35 (0.26-2.43) | 2.01 (0.81-3.21) | 2.68 (1.25-4.11) | 3.35 (1.62-5.08) |
|  | **5 years** | 1.05 (-0.07-2.16) | 1.58 (0.49-2.67) | 2.25 (1.06-3.44) | 2.92 (1.5-4.33) | 3.58 (1.88-5.29) |
|  | **10 years** | 1.34 (0.18-2.5) | 1.88 (0.76-3.0) | 2.54 (1.34-3.75) | 3.21 (1.8-4.62) | 3.88 (2.19-5.57) |
|  | **15 years** | 1.64 (0.41-2.86) | 2.17 (1.0-3.35) | 2.84 (1.6-4.08) | 3.51 (2.08-4.93) | 4.18 (2.48-5.87) |
|  | **20 years** | 1.93 (0.62-3.25) | 2.47 (1.21-3.72) | 3.13 (1.84-4.43) | 3.8 (2.34-5.27) | 4.47 (2.76-6.18) |
|  | **25 years** | 2.23 (0.81-3.64) | 2.76 (1.41-4.11) | 3.43 (2.05-4.81) | 4.01 (2.58-5.62) | 4.77 (3.02-6.52) |
|  | **30 years** | 2.52 (0.99-4.05) | 3.06 (1.6-4.51) | 3.73 (2.26-5.19) | 4.39 (2.8-5.98) | 5.07 (3.26-6.86) |
|  | **35 years** | 2.82 (1.16-4.47) | 3.35 (1.78-4.93) | 4.02 (2.45-5.6) | 4.69 (3.01-6.37) | 5.36 (3.49-7.22) |
|  | **40 years** | 3.11 (1.33-4.9) | 3.65 (1.94-5.35) | 4.32 (2.62-6.01) | 4.98 (3.21-6.76) | 5.65 (3.7-7.6) |
| **ST-junction** | **1 year** | 0.26 (-0.45-0.98) | 1.03 (0.31-1.75) | 1.99 (0.97-3.01) | 2.95 (1.49-4.41) | 3.91 (1.96-5.86) |
|  | **5 years** | 0.54 (-0.12-1.19) | 1.31 (0.64-1.97) | 2.27 (1.29-3.25) | 3.23 (1.79-4.66) | 4.19 (2.26-6.11) |
|  | **10 years** | 0.88 (0.27-1.5) | 1.65 (1.03-2.27) | 2.61 (1.65-3.57) | 3.57 (2.15-4.99) | 4.53 (2.61-6.45) |
|  | **15 years** | 1.23 (0.61-1.85) | 1.99 (1.36-2.62) | 2.95 (1.99-3.92) | 3.91 (2.49-5.34) | 4.87 (2.95-6.8) |
|  | **20 years** | 1.57 (0.9-2.24) | 2.34 (1.66-3.02) | 3.3 (2.3-4.3) | 4.26 (2.81-5.71) | 5.22 (3.28-7.16) |
|  | **25 years** | 1.91 (1.15-2.68) | 2.68 (1.91-3.45) | 3.64 (2.58-4.7) | 4.6 (3.11-6.1) | 5.56 (3.59-7.54) |
|  | **30 years** | 2.26 (1.38-3.14) | 3.03 (2.14-3.91) | 3.99 (2.84-5.13) | 4.95 (3.39-6.5) | 5.91 (3.88-7.93) |
|  | **35 years** | 2.6 (1.6-3.61) | 3.37 (2.36-4.39) | 4.33 (3.08-5.58) | 5.29 (3.66-6.93) | 6.25 (4.16-8.34) |
|  | **40 years** | 2.95 (1.8-4.1) | 3.71 (2.56-4.87) | 4.67 (3.31-6.04) | 5.63 (3.91-7.36) | 6.59 (4.44-8.75) |
| **Asceding aorta** | **1 year** | -0.9 (-1.71- -0.85) | -0.49 (-1.26-0.28) | 0.02 (-1.0-1.05) | 0.54 (-0.92 -1.99) | 1.05 (-0.9-2.99) |
|  | **5 years** | -0.37 (-1.14- -0.4) | 0.04 (-0.7 0.77) | 0.55 (-0.46-1.56) | 1.06 (-0.39-2.51) | 1.57 (-0.38-3.52) |
|  | **10 years** | 0.29 (-0.45-1.03) | 0.7 (-0.27-1.42) | 1.21 (0.19-2.23) | 1.72 (0.25-3.19) | 2.23 (0.26-4.2) |
|  | **15 years** | 0.95 (0.2-1.7) | 1.36 (0.6-2.11) | 1.87 (0.81-2.92) | 2.38 (0.87-3.88) | 2.89 (0.89-4.89) |
|  | **20 years** | 1.61 (0.81-2.4) | 2.01 (1.2-2.83) | 2.53 (1.41-3.64) | 3.04 (1.48-4.59) | 3.55 (1.5-5.6) |
|  | **25 years** | 2.26 (1.4-3.13) | 2.67 (1.77-3.57) | 3.18 (1.99-4.38) | 3.69 (2.07-5.32) | 4.21 (2.1-6.32) |
|  | **30 years** | 2.92 (1.96-3.89) | 3.33 (2.32-4.34) | 3.84 (2.55-5.13) | 4.35 (2.65-6.06) | 4.86 (2.68-7.04) |
|  | **35 years** | 3.58 (2.5-4.66) | 3.99 (2.86-5.12) | 4.5 (3.1-5.9) | 5.01 (3.22-6.81) | 5.52 (3.26-7.78) |
|  | **40 years** | 4.24 (3.03-5.44) | 4.65 (3.39-5.91) | 5.16 (3.64-6.67) | 5.67 (3.77-7.57) | 6.18 (3.83-8.53) |
